# Utilization of the Public Health Ordinance to prevent nosocomial spread in a case of acute measles-associated psychosis

**DOI:** 10.1186/s13584-020-00435-9

**Published:** 2021-01-15

**Authors:** Chen Stein-Zamir, Nitza Abramson, Hagit Sonnenfeld-Alroey, Jacob Charnes, Dana Eckstein, Aryeh Dienstag, Dana Wolf, Allon E. Moses, Yoram G. Weiss

**Affiliations:** 1 Jerusalem District Health Office, Ministry of Health, Jerusalem, Israel; 2 The Hebrew University of Jerusalem, Faculty of Medicine, Braun School of Public and Community Medicine, Jerusalem, Israel; 3 Department of Neurology, Hadassah Hebrew University School of Medicine, Jerusalem, Israel; 4 Department of Psychiatry, Hadassah Hebrew University School of Medicine, Jerusalem, Israel; 5 Department of Clinical Microbiology and Infectious Diseases, Hadassah Hebrew University School of Medicine, Jerusalem, Israel; 6 Central Administration, Hadassah Hebrew University School of Medicine, Jerusalem, Israel

**Keywords:** Measles, The public health ordinance, Measles complications, Nosocomial infection, Isolation

## Abstract

Measles is a highly contagious disease. A 24 years old patient, recently exposed to measles (unvaccinated), presented in the emergency department with severe agitation, compatible with an acute psychotic episode, during the measles epidemic which spread in Israel in 2018–2019. Upon hospital admission, strict isolation was instructed, yet, without compliance, probably due to the patient’s status. Measles diagnosis was promptly confirmed. As measles transmission was eminent, public health measures were employed through immediate implementation of the section 15 of the Public Health Ordinance, allowing for compulsory short-term isolation. The patient’s condition improved within a few days and the measures were no longer necessary. This measles case occurred in the pre-Coronavirus disease 2019 (COVID-19) epidemic when use of a Public Health Ordinance was considered an extreme measure. This is in contrast to the current global use of Public Health laws to enforce strict quarantine and isolation on persons infected or potentially exposed to COVID-19. Nevertheless, minimizing infectious diseases transmission is a core function of public health law. Utilizing legal enforcement in circumstances of immediate public health hazard, such as nosocomial measles transmission, necessitates careful consideration. The integrative clinical and public health approach and prompt measures employed in this exceptional case, led to prevention of further infection spread.

## Key points

- ○ Since 2017, recurrent measles outbreaks emerged in many countries with international spread.
- ○ Nosocomial measles spread should be avoided due to exposure risk endangering highly susceptible individuals.
- ○ Public health enforcement in eminent cases of nosocomial measles spread should be implemented prudently.
- ○ Public health enforcement is no longer novel in the COVID-19 epidemic era.
- ○ Yet, the increasing vaccine hesitancy in developed countries, including Israel, may present a threat for future epidemics and need for public health actions.

## Introduction

Measles is one of the most contagious infectious diseases in humans. A measles case may potentially infect, on average, 12–18 individuals in an entirely susceptible population [1]. Measles is transmitted via person-to-person contact by airborne spread. The incubation period is 7–21 days (median 14 days). The period of contagiousness is considered from 4 days before the rash to 4 days afterwards [1, 2].

Measles is a vaccine-preventable-disease for decades; the recommended prevention strategy includes two measles vaccine doses. Accordingly, the routine immunization schedule in Israel includes two doses of MMRV (Measles-Mumps-Rubella-Varicella) vaccine at 12 months and 6 years. Despite progress towards measles elimination during 2000–2016, an increase in incidence and mortality occurred since 2017; measles outbreaks emerged in many countries with international dissemination [3]. During 2018–2019, a large measles outbreak spread in Israel (probably post importation) with 4300 confirmed cases. Half the cases occurred in the Jerusalem district, mainly unvaccinated children and adolescents in Jewish ultra-orthodox communities [4, 5].

Measles is a notifiable disease in Israel by law; cases are reported to the district health office. Case definition includes clinical, laboratory and epidemiologic characteristics. Laboratory confirmation includes reverse transcription–polymerase chain reaction (PCR) testing or detection of measles immunoglobulin M (IgM). Epidemiologic investigation includes demographic, clinical and laboratory data, date of disease onset, immunization status, hospitalization, and cases in the household. The ministry of health guidelines demand that all health providers employ immediate strict isolation of patients with a febrile rash illness evaluated for measles. Upon admission, suspected cases should be sampled by throat or nasopharyngeal swabs and urine for measles PCR and serum for measles IgM. The health providers should ensure all staff are up-to-date on MMR vaccine and other vaccinations.

## Case report

During the measles outbreak in Jerusalem, a 24 years old male student presented at the emergency department with marked agitation and disorientation.

The patient resided in a Jerusalem ultra-orthodox neighborhood. He had travelled for religious purposes to Uman, Ukraine 3 weeks earlier, with exposure to measles during the travel. Two weeks after his return, he developed conjunctivitis, rash and mild fever, lasting for 2–3 days and then accompanied by increasing agitation. Medical history included mild hemiparesis, subsequent to injury in an accident a decade earlier. Family history was positive for schizoaffective disorder. No intake of medications or other substances had been reported. The patient had not been vaccinated with measles vaccine previously.

Upon admission the patient displayed agitation, confusion, fast and disorganized speech, bizarre behavior and disorientation. Vital signs included blood pressure 125/85 mmHg, heart rate 80 beats/minute, oxygen saturation 95% and body temperature 37.9 °C. Physical examination showed mild spastic right hemiparesis, without further abnormalities.

Laboratory workup included blood count with hemoglobin 13.9 g/dl, white blood cells (WBC) 13.6 × 10^9^/L (65% neutrophils), and platelet count 160 × 10^9^/L. C-reactive protein 2.6 mg/dl (normal range 0–5 mg/dl). A lumbar puncture revealed normal pressure, WBC count of 8 lymphocytes and 2 polymorphonuclear leukocytes, CSF protein and glucose concentration were normal. The PCR tests results for Herpes viruses and Enteroviruses were negative. The CT imaging showed left frontotemporal damage (probably linked to past injury). EEG showed mild diffuse slowing, periods of generalized slowing accentuated over the left frontotemporal region.

The patient was admitted to the neurology department with suspected measles-associated CNS complications. He was severely agitated; despite repeating instructions to keep the relevant isolation instructions, he continued wandering in the department and in other hospital wards. The patient was potentially highly infectious, in contact with vulnerable persons and unable to comply with isolation instructions. Hence, the hospital’s director informed the district health officer requesting implementation of legal authority. In order to apply legal actions, laboratory confirmation was obligatory. Confirmation included positive measles PCR in both throat swab and urine sample, indicating active infectivity. Measles PCR of CSF was subsequently positive. Serology showed positive measles IgM and IgG.

After discussions with neurology, infectious diseases and legal specialists, the district health officer issued an immediate order based on section 15 of the Public Health Ordinance. The order included instructions to restrict the patient to an isolated room and allow use of security supervision to ensure compliance. All personnel caring for the patient had to be fully immunized (the Ministry of Health directives mandate two measles vaccine doses with proper documentation, for all health workers).

Twelve hours later the Magistrate Court evaluated and confirmed the order. The court re-confirmed the order on the next day, in the presence of the patient’s legal representative, pending a re-evaluation within 3 days. Over the next days the patient’s condition and co-operation improved significantly, the order was no longer necessary and accordingly waived.

## Discussion

Previous application of the section 15 of the Public Health Ordinance in Israel had been utilized in isolation of active tuberculosis cases. To our knowledge, this is its first use as a public health measure in a measles case. The Public Health Ordinance (1940) was formerly part of the British mandate law and updated since. The ordinance makes provisions for health regulation applying to routine (e.g. mandatory notification on diseases and mortality, environmental sanitation, water quality) along with epidemic circumstances [6, 7].

A core element of health legal enforcement is the obligation to balance between individual rights and collective good, in the complex management of infectious diseases’ hazards. Immediate response towards measles as a public health emergency is warranted, due to the high transmission risk. Appling stringent and prompt isolation measures is crucial in hospitals, caring for immune-compromised and at-risk patients, to prevent nosocomial spread. Measles nosocomial transmission is a significant and emerging option of infection spread, even in countries where measles is otherwise under control. Health-care settings play a critical role in measles transmission and generation of secondary cases [8, 9].

The measles case occurred in the pre-Coronavirus disease 2019 (COVID-19) epidemic when use of a Public Health Ordinance was considered an extreme measure. This is in stark contrast to the current worldwide use of Public Health laws to enforce strict quarantine and isolation on persons infected or potentially exposed to COVID-19 (https://www.lawfareblog.com/quarantine-and-isolation-authorities-states-affected-covid-19; https://www.bloomberglaw.com/product/health/document/XCSD21H4000000; https://www.kingsleynapley.co.uk/insights/blogs/criminal-law-blog/covid-19-the-legal-basis-for-quarantine). Nevertheless, minimizing infectious diseases transmission is a core function of public health law. Law also has a role in allowing public health authorities to limit contact with infectious individuals and to exercise emergency powers in outbreak settings [10, 11]. In the USA large-scale isolation and quarantine was last enforced during the 1918–1919 influenza (“Spanish Flu”) pandemic (https://www.cdc.gov/quarantine/aboutlawsregulationsquarantineisolation.html).

Measles cases of Israel’s 2018–2019 outbreak were mainly unvaccinated young persons [5]. Some 8–10% required hospitalization. The main complications were pneumonia/pneumonitis, diarrhea and otitis media. Neurological and psychiatric complications were uncommon, and included meningitis, encephalitis, visual disturbances, dysarthria, behavioural alternations and chorea [4]. The measles virus may persist in the brain for years, triggering fatal neurodegenerative diseases as subacute sclerosing panencephalitis (SSPE) and measles inclusion-body encephalitis [12]. Our case is a young adult, presenting with measles-related neuropsychiatric manifestations consistent with acute psychosis (manic episode), which resolved within days. Behavioral disturbances in measles probably stem from encephalitis or triggering of an occult psychiatric disorder. Clinical presentation of measles as acute psychosis is indeed uncommon, even though the virus is highly neurotropic.

## Conclusions

Utilizing legal enforcement in circumstances of immediate public health hazard, such as nosocomial measles transmission, necessitates careful consideration. The integrative clinical and public health approach and prompt measures employed in this exceptional case, led to prevention of further infection spread.
